# Inactive DNMT3B Splice Variants Modulate *De Novo* DNA Methylation

**DOI:** 10.1371/journal.pone.0069486

**Published:** 2013-07-19

**Authors:** Catherine A. Gordon, Stella R. Hartono, Frédéric Chédin

**Affiliations:** Department of Molecular and Cellular Biology, University of California Davis, Davis, California, United States of America; Florida State University, United States of America

## Abstract

Inactive DNA methyltransferase (DNMT) 3B splice isoforms are associated with changes in DNA methylation, yet the mechanisms by which they act remain largely unknown. Using biochemical and cell culture assays, we show here that the inactive DNMT3B3 and DNMT3B4 isoforms bind to and regulate the activity of catalytically competent DNMT3A or DNMT3B molecules. DNMT3B3 modestly stimulated the *de novo* methylation activity of DNMT3A and also counteracted the stimulatory effects of DNMT3L, therefore leading to subtle and contrasting effects on activity. DNMT3B4, by contrast, significantly inhibited *de novo* DNA methylation by active DNMT3 molecules, most likely due to its ability to reduce the DNA binding affinity of co-complexes, thereby sequestering them away from their substrate. Immunocytochemistry experiments revealed that in addition to their effects on the intrinsic catalytic function of active DNMT3 enzymes, DNMT3B3 and DNMT34 drive distinct types of chromatin compaction and patterns of histone 3 lysine 9 tri-methylation (H3K9me3) deposition. Our findings suggest that regulation of active DNMT3 members through the formation of co-complexes with inactive DNMT3 variants is a general mechanism by which DNMT3 variants function. This may account for some of the changes in DNA methylation patterns observed during development and disease.

## Introduction

Mammalian cytosine methylation is a key epigenetic modification that is maintained upon cell division and thus represents a form of cellular memory. It is involved in regulation of gene expression, silencing of parasitic transposable elements, X-chromosome inactivation, genomic imprinting, the maintenance of genomic stability, and is consistently altered in human cancers [1], [2], [3]. Three DNA methyltransferases (DNMTs) actively catalyze the transfer of methyl groups to CpG sites in mammals, including DNMT1, DNMT3A, and DNMT3B [2]. DNMT3A and DNMT3B primarily act as *de novo* enzymes, setting up the initial patterns of DNA methylation during early embryonic development, while DNMT1 primarily acts as a maintenance DNMT, copying DNA methylation patterns upon cell division [4], [5]. In addition to these three active enzymes, a catalytically inactive DNMT3 variant, the DNMT3-Like protein, DNMT3L, plays an essential role in *de novo* DNA methylation [6]. DNMT3L operates by binding to and reorganizing large heterogeneous DNMT3A or DNMT3B complexes into defined heterodimeric DNMT3A or DNMT3B:DNMT3L sub-complexes with higher affinity for S-Adenosyl-L-Methionine (SAM), increased catalytic output, and higher processivity [7], [8], [9], [10], [11], [12], [13].

Interestingly, over thirty different isoforms of DNMT3B, resulting from alternative splicing and/or alternative promoter usage, have been reported [14], [15], [16], [17]. Splicing patterns and spatio-temporal patterns of isoform expression appear to be conserved between humans and mice, suggesting that these isoforms carry biological significance [18]. Despite this conservation, their functions in normal and disease states remain largely unclear. Several of these DNMT3B variants are missing key regions of their C-terminal catalytic domain, rendering them catalytically inactive. Some of these variants were reported to be overexpressed in various cancers and to be associated with global changes in DNA methylation [14], [15], [16], [17], [18], [19], [20], [21]. For instance, overexpression of DNMT3B7, a major splice variant found in numerous tumor types, triggers both global and local changes in DNA methylation patterns as well as chromosomal rearrangements [16], [22]. Likewise, DNMT3B3Δ5 is upregulated in liver and skin cancer cell lines and its overexpression in HCT116 cells results in loss of DNA methylation at centromeric and pericentromeric repetitive elements [17]. Though the expression of inactive DNMT3B variants is associated with changes in DNA methylation, little is known about the mechanisms that underlie these changes at the molecular level.

In the present study, we sought to better understand how inactive DNMT3B variants are involved in DNA methylation, focusing on two inactive DNMT3B variants. The DNMT3B3 isoform, which lacks all sequences located between catalytic motifs VIII and IX, including the first nine amino acids of catalytic motif IX (Figure S1A), is ubiquitously expressed in normal human tissues [23]. Intriguingly, the expression pattern of *DNMT3B* is shifted from DNMT3B1 to DNMT3B3 immediately upon differentiation of human embryonic stem (ES) cells [24]. Expression analyses show that DNMT3B3 is overexpressed in numerous cancerous tissues and tumor cell lines [25], and its expression was reported to be essential for cancer cell survival [26]. DNMT3B3 has been shown to either contribute to DNA methylation activity or to be associated with the ability to maintain aberrant DNA methylation patterns in cancer [25],[26],[27]. The possible involvement of DNMT3B3 in the deposition and/or maintenance of DNA methylation patterns is intriguing given that it is catalytically inactive [19],[21],[28]. The DNMT3B4 isoform corresponds to a truncated DNMT3B variant lacking exons 21–23 encompassing all residues located downstream of conserved motif XIII [14] (Figure S1A). DNMT3B4 is expressed in multiple tissues in mouse [23], but appears to be overexpressed in hepatocellular carcinomas (HCCs), for which DNMT3B4 overexpression is significantly correlated with DNA hypomethylation at pericentric satellite repeats [20]. In chronic myeloid leukemia, increased levels of DNMT3B4 are associated with *LINE1* DNA hypomethylation [29]. In addition, HEK293 cell lines that stably overexpress DNMT3B4 display demethylation of satellite 2 after multiple rounds of passages [20]. This suggests that overexpression of DNMT3B4 somehow induces loss of DNA methylation.

Here, we used DNMT3L as a paradigm for the regulation of *de novo* DNMTs by inactive variants and tested the hypothesis that inactive DNMT3B isoforms can form complexes with, and modulate the activity of, catalytically competent DNMT3B or DNMT3A isoforms. Our data shows that DNMT3B3 binds to catalytically competent DNMT3 molecules and modulates their catalytic activity. DNMT3B4 also binds to catalytically competent DNMT3 molecules; however complex formation leads to a strong reduction in DNA binding and DNMT activity, causing a dominant-negative inhibition. Our results suggest that binding and regulation of active DNMT3 variants by inactive DNMT3B variants is a general mechanism accounting, at least in part, for some of the changes in DNA methylation profiles observed in normal and disease states.

## Materials And Methods

### Expression Vectors

Mammalian expression vectors carrying N-terminally Myc- and FLAG- tagged human *DNMT3B1*, *DNMT3B2*, *DNMT3B3*, *DNMT3A1*, *DNMT3A2*, and *DNMT3L* coding regions have been previously described [12],[21]. The human *DNMT3B4* cDNA was created by a combination of restriction enzyme cloning and PCR (primers available upon request) using pcDNA3/Myc-DNMT3B2 as a template, and was verified by sequencing. Expression vectors for bacterial expression include a modified pGEX vector [12], carrying an N-terminal glutathione S-transferase (GST) tag followed by a 6X histidine tag prior to full-length or C-terminal *DNMT3* isoform cDNA; and a modified pMAL vector (NEB) containing an N-terminal maltose-binding protein (MBP) tag followed by a 6X histidine tag prior to full-length or C-terminal *DNMT3* isoform cDNA. Vectors were created by restriction enzyme cloning and PCR, and verified by sequencing. C-terminal *DNMT3B2*, *DNMT3B3*, and *DNMT3B4* isoforms were missing the coding region for their first 510 amino acids, and therefore encoded for proteins that were 323, 260, and 214 amino acids long, respectively. Note that C-terminal *DNMT3B2* is equivalent to C-terminal *DNMT3B1* since *DNMT3B2* and *DNMT3B1* only differ in their N-terminus. C-terminal *DNMT3B* isoforms were codon-optimized for bacterial expression.

### Episomal DNA

The pFC19 episome [7] was used as a DNA methylation target. The episome contains the EBNA1/OriP replication system and can be stably maintained in mammalian cells. The regions analyzed for DNA methylation correspond to ∼500 base pair region of the pBR322 backbone, carrying 48 CpG sites [30] and a 1 kb region of the human *SNRPN* CpG island.

### Protein Purification

Full-length human DNMT3A2 was purified as described previously [12]. Human MBP-6X histidine-tagged full-length DNMT3L (MBP-3L), C-terminal DNMT3B2 (MBP-3B2ct), C-terminal DNMT3B3 (MBP-3B3ct), and C-terminal DNMT3B4 (MBP-3B4ct) proteins were purified from freshly transformed *Escherichia coli* (*E. coli)* Rosetta (DE3) cells (Novagen) grown in LB broth supplemented with ampicillin (100 µg/ml) and 0.2% glucose. Protein expression was induced during exponential growth with 0.5 mM isopropyl 1-thio-β-D-galactopyranoside and the culture was incubated at 16°C overnight. Cells were harvested and lysed in chilled amylose buffer (20 mM Tris-HCl pH 7.5, 150 mM NaCl, 0.1 mM EDTA, 0.1 mM dithiothreitol (DTT), 10 µg/ml RNase A, 0.5 mM phenylmethylsulfonyl fluoride, 0.1% Triton X-100, and 1 complete, Mini, EDTA-free, protease inhibitor cocktail tablet (Roche) using a microfluidizer. The lysate was spun for one hour at 33,000×*g* to remove cellular debris and the supernatant was subsequently loaded onto a 30 ml amylose column. The column was washed with at least three column volumes of amylose buffer and then bound protein eluted with amylose buffer supplemented with 20 mM maltose. For MBP-3B2ct, amylose elutions were dialyzed overnight in a low salt buffer (20 mM Tris-HCl pH 7.5, 50 mM NaCl, 0.1 mM EDTA, and 0.1 mM DTT) and then loaded onto a 1 ml Q column (GE HealthCare) followed by a 1 ml SP column (GE Healthcare). The flow-through of the Q and SP columns was then loaded onto a 5 ml heparin column (GE Healthcare), which was eluted with a salt gradient up to 1.5 M NaCl after washing. MBP-3L was purified as described for MPB-3B2ct except the SP column was omitted. MBP-3B3ct was purified as described for MBP-3B2ct except the heparin column was omitted. For MBP-3B4ct, amylose elutions were loaded directly onto a heparin column after dialysis in low salt buffer. After washing, bound protein was eluted using a salt gradient as described. Heparin elutions were then dialyzed in S200 buffer (20 mM Tris-HCl pH 7.5, 500 mM NaCl, 0.5 mM EDTA, and 0.5 mM DTT) and run through an S200 size exclusion column.

C-terminal and full-length co-complexes of MBP-6X histidine tagged DNMT3B2 (MBP-3B2ct or MBP-3B2) with GST-6X histidine tagged DNMT3B2 (GST-3B2ct or GST-3B2), DNMT3B3 (GST-3B3ct or GST-3B3), or DNMT3B4 (GST-3B4ct or GST-3B4) were obtained by overexpressing differentially tagged DNMT3B isoforms in *E. coli* individually, and lysing appropriate pairwise combinations of cells together. The clarified lysates were then run through a 10 ml amylose column followed by a 5 ml glutathione column. Growth, expression, lysis and amylose column conditions were essentially as described above. Elutions from the amylose column were dialyzed in GST binding buffer (1 mM EDTA, 1 mM DTT, and 1X phosphate buffered saline) overnight and loaded onto a 5 ml glutathione column, which was washed with at least five column volumes of GST wash buffer (50 mM Tris-HCl pH 8.5, 500 mM NaCl, 1 mM EDTA, 1mM DTT, and 0.01% Triton X-100) and eluted with GST wash buffer supplemented with 4 mM reduced glutathione. The resulting elutions contained co-complexes of GST and MBP-tagged DNMT3B isoforms.

Protein concentrations were determined by either a Bradford assay and/or by their absorbance at 280 nm, using their molecular weights and extinction coefficients. All purified proteins and protein complexes were stored at −80°C in storage buffer (20 mM Tris-HCl pH 7.5, 150 mM NaCl, 0.1 mM EDTA, 0.1 mM DTT, 40% glycerol).

### Cell Lines, Transfections And Immunocytochemistry

HEK293, HEK293c18, and NIH3T3 cell lines were grown under standard conditions. Transient transfections for western blots, immunoprecipitation experiments, or immunofluorescence experiments were performed in either 6-well or 10 cm plates, using either 0.5 µg or 2.5 µg of expression vectors, respectively, unless otherwise specified. For immunoprecipitation experiments and most western blots, cells were harvested 36 to 48 hours post transfection by whole cell extract sonication lysis. The histone enriched fraction was harvested by the acid extraction method. Transfections for *in vivo* DNA methylation analysis were performed in 6-well plates with 0.5 µg of expression vector and 0.5 µg of episomal DNA, as described before [30]. All transfections were performed at least in duplicate, using calcium phosphate, Lipofectamine (Invitrogen), or Turbofect (Fermentas) methods.

Immunocytochemistry on transfected HEK293 cells was performed on gelatinized glass coverslips using the common formaldehyde/Triton X-100 fixation and permeabilization method. Primary antibodies (Mouse anti-FLAG: 1∶150, Rabbit anti-Myc: 1∶100, Rabbit anti-H3K9me3∶1:1000 (Abcam) were incubated for one hour, washed three times and revealed using fluorophore-conjugated secondary antibodies (1∶200). After further washing, coverslips were mounted on glass slides in a drop of Vectashield Mounting Medium with DAPI (4′, 6-diamidino-2-phenylindole), and imaged using a fluorescence microscope.

### Measurement Of DNA Methylation Activity *in vitro* and *in vivo*

*In vitro* DNA methylation patterns laid by full-length DNMT3B isoform co-complexes were determined by bisulfite methylation sequencing upon incubation of 1 µM purified recombinant MBP and GST tagged co-complexes with 250 ng of pFC19 DNA template in the presence of 100 µM S-Adenosyl-L- methionine (SAM) in activity buffer (25 mM Tris-HCl pH 7.5, 50 mM KCl, 0.5 mM MgCl_2_, 100 µg/ml BSA, 1 mM DTT). The reactions were incubated for 24 hours at 37°C (SAM was re-supplemented fresh after 12 hours). After the 24 hour reaction, proteins were removed by Proteinase K treatment followed by phenol chloroform extraction and ethanol precipitation. *In vitro* DNA methyltransferase activity was quantitated by measuring the transfer of tritiated methyl groups from S-adenosyl-L-[methyl- H^3^] methionine (H^3^-SAM) onto double stranded poly (dIdC) or pFC19 DNA substrates as described previously [12]. *In vivo* DNA methylation of the pFC19 target episome in HEK293c18 cells expressing various DNMT3B isoforms was assessed by Southern blots and bisulfite methylation sequencing as described previously [30].

### Electromobility Shift Assays

DNA binding reactions were set up in activity buffer (25 mM Tris-HCl pH 7.5, 50 mM KCl, 0.5 mM MgCl_2_, 100 µg/ml bovine serum albumin, 1 mM DTT) supplemented with 10% glycerol. Purified proteins were pre-incubated at varying concentrations for 10 minutes at 37°C before addition of 0.1 µM of human DNA (a 420 base pair DNA fragment of the PWWP domain of human DNMT3B described previously [10]). DNA binding was allowed to reach equilibrium over 30 minutes at 37°C, at which point samples were loaded onto a 1% agarose gel and run at 65 V for 1.5 hours. DNA binding was visualized by ethidium bromide staining of the DNA fragment and analyzed via ImageQuant software version 5.2. DNA binding reactions were performed at least in duplicate.

### Immunoprecipitation Experiments

Immunoprecipitation experiments were done as described previously [21]. Briefly, pCDNA3/FLAG-DNMT3B1 was transiently co-transfected with expression constructs for Myc-tagged DNMT3B1, DNMT3B2, DNMT3B3, or DNMT3B4 isoforms. After harvesting the cells, lysates were incubated with anti-FLAG M2 affinity gel (Sigma) with rotation for three hours at 4°C. After washing five times, proteins bound to the affinity gel were eluted by competition with the FLAG peptide, separated by 8% SDS-PAGE gels, and transferred to PVDF membranes. Thereafter, western blots were performed with anti-FLAG (Sigma) and anti-Myc (Roche) antibodies.

### MBP Pull-downs

Purified MBP-tagged DNMT3B3ct (0.8 µM) and MBP-tagged DNMT3L (0.8 µM) were each incubated with purified full-length DNMT3A2 (0.8 µM) at 37°C for one hour in binding buffer (25 mM Tris-HCl pH 7.5, 50 mM KCl, 0.5 mM MgCl_2_, 1 mM DTT). 50 µl of amylose resin (NEB) was added to the protein co-complexes and proteins were incubated an additional hour at 4°C with gentle rocking. Co-complexes were washed three times in binding buffer and then eluted with binding buffer supplemented with 20 mM maltose. The resulting elutions were loaded on 8% SDS-PAGE gels and visualized after electrophoresis by staining with Coommassie brilliant blue.

### Gel Filtration

100 µg of purified DNMT3B C-terminal proteins and C-terminal DNMT3B isoform co-complexes were loaded onto a Superose 6 HR 10/30 column controlled by an ÄKTA*FPLC* system run by Unicorn version 4.00.16 software (GE Healthcare). Superose 6 buffer (20 mM Tris-HCl pH 7.5, 150 mM NaCl, 0.1 mM EDTA, 0.1 mM DTT) was used for all experiments and flow rate was kept constant at 0.3 ml/minute. Elution volumes were determined using Unicorn software.

## Results

### DNMT3B3 and DNMT3B4 Interact with Active DNMT3 Proteins

**Figure 1:**
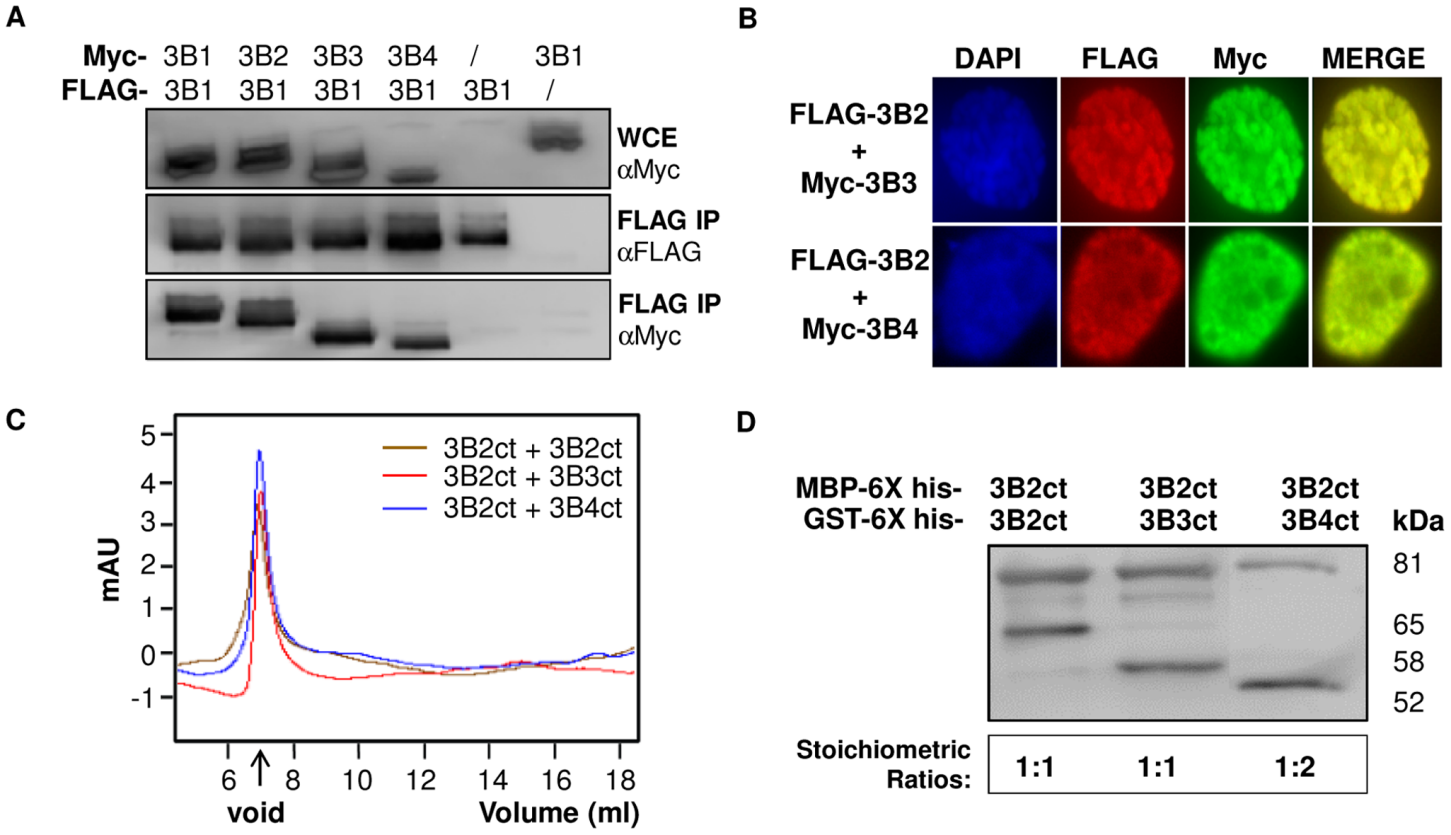
Inactive DNMT3B isoforms interact with active DNMT3 isoforms. (A) Combinations of Myc- and FLAG- tagged isoforms were expressed in human HEK293 cells as indicated. Expression of Myc-tagged proteins was verified in the whole cell extract (WCE) by western blot (top). FLAG immunoprecipitates (IP) were probed with FLAG antibody to verify the expression of FLAG-tagged DNMT3B1 (center). Myc-tagged proteins associated with FLAG IP were revealed using an anti-Myc antibody (bottom). (B) Myc-tagged DNMT3B3 or Myc-tagged DNMT3B4 and FLAG-tagged DNMT3B2 were expressed in HEK293 cells and their localization was analyzed by immunofluorescence as indicated. All DNMT3B2 and DNMT3B3 dual-expressing cells showed co-localization of expression (100%; n = 49) and most DNMT3B2 and DNMT3B4 dual-expressing cells displayed co-localization (96.2%; n = 52) Representative images of are shown. (C) Representative elution profiles from gel filtration chromatography are shown revealing that DNMT3B co-complexes form high molecular weight aggregates indistinguishable in size. (D) Co-complexes between indicated isoforms were purified through tandem affinity purification and the relative stoichiometry of each isoform in the complex was evaluated by western blot using an anti-6X histidine antibody directed against the internal 6X histidine tag common to all isoforms. A representative blot is shown and relative stoichiometric ratios are indicated below. Experiments were performed at least in triplicate.

We first tested whether DNMT3B3 and DNMT3B4 could bind to active DNMT3B1 and/or DNMT3B2 enzymes (see Figure S1A for a schematic of all DNMT3 variants used here). To determine interaction patterns, we used a cell culture model and expressed full-length FLAG-tagged DNMT3B1 with Myc-tagged DNMT3B3 or DNMT3B4 in human HEK293 cells and performed co-immunoprecipitation experiments. These assays revealed that DNMT3B3 and DNMT3B4 physically interact with DNMT3B1 (Figure 1A). To gain further evidence for this interaction, we tested whether DNMT3B2 and DNMT3B3 or DNMT3B4 isoforms co-localize in HEK293 cells. For this, we co-expressed FLAG-tagged DNMT3B2 with Myc-tagged DNMT3B3 or DNMT3B4, and performed immunofluorescence experiments. As expected, DNMT3B2 co-localized with DNMT3B3 and DNMT3B4 in the nucleus of cells expressing both proteins (Figure 1B), further suggesting that active and inactive DNMT3B proteins physically interact *in vivo*.

We next tested whether DNMT3B3 and DNMT3B4 could bind to active DNMT3B molecules *in vitro.* For this, we created expression vectors for C-terminal or full-length DNMT3B1, DNMT3B2, DNMT3B3, and DNMT3B4 isoforms, carrying either an N-terminal MBP tag or an N-terminal GST tag. DNMT3B isoforms were expressed recombinantly in *E. coli*, and appropriate combinations of cells expressing differentially tagged DNMT3B isoforms were lysed together and co-complexes purified through sequential amylose and glutathione affinity columns. Using this technique, we were able to show that DNMT3B2 (both full-length and C-terminal versions) formed co-complexes with itself as well as with DNMT3B3 and DNMT4B4 (Figure S1B). Using MBP pull-down assays, we also showed that DNMT3B3 could interact with full-length DNMT3A2 protein at least as well as DNMT3L under the same condition (data not shown). To reveal the stoichiometry of each subunit within co-complexes, we first used gel filtration chromatography: each C-terminal co-complex eluted from a Superose 6 column at the void volume, indicating that they form large molecular weight complexes (Figure 1C). To determine the oligomerization profile of each isoform individually, we purified MBP-tagged, C-terminal versions of DNMT3B2, DNMT3B3, and DNMT3B4 (see Materials and Methods for purification scheme and Figure S1C for protein purity), and ran 100 µg of each purified protein on a Superose 6 column. As observed for co-complexes, each single isoform existed as soluble high molecular weight aggregates eluting from Superose 6 at the void volume (data not shown). This was surprising given that DNMT3L, which resembles DNMT3B3 and DNMT3B4 (see Figure S1A), commonly forms monomer or dimers in solution [11],[12]. In order to gain insight into protein stoichiometry within co-complexes, we used an anti-6X histidine antibody (which lights up each fusion protein within co-complexes) and western blot to quantify the intensities of each band. This showed that DNMT3B2:DNMT3B2 and DNMT3B2:DNMT3B3 co-complexes exist in roughly 1∶1 stoichiometries while DNMT3B2:DNMT3B4 co-complexes show a roughly 1∶2 ratio of DNMT3B2 to DNMT3B4 (Figure 1D). Thus, even though each co-complex corresponds to a large high molecular weight aggregate, it appears that each co-complex has a precise subunit stoichiometry. Taken together, this data indicates that inactive DNMT3B isoforms bind to active DNMT3 members and form large molecular weight complexes of defined stoichiometries.

### DNMT3B3 Associates with Active DNA Methylation without Modifying DNA Methylation Patterns

**Figure 2:**
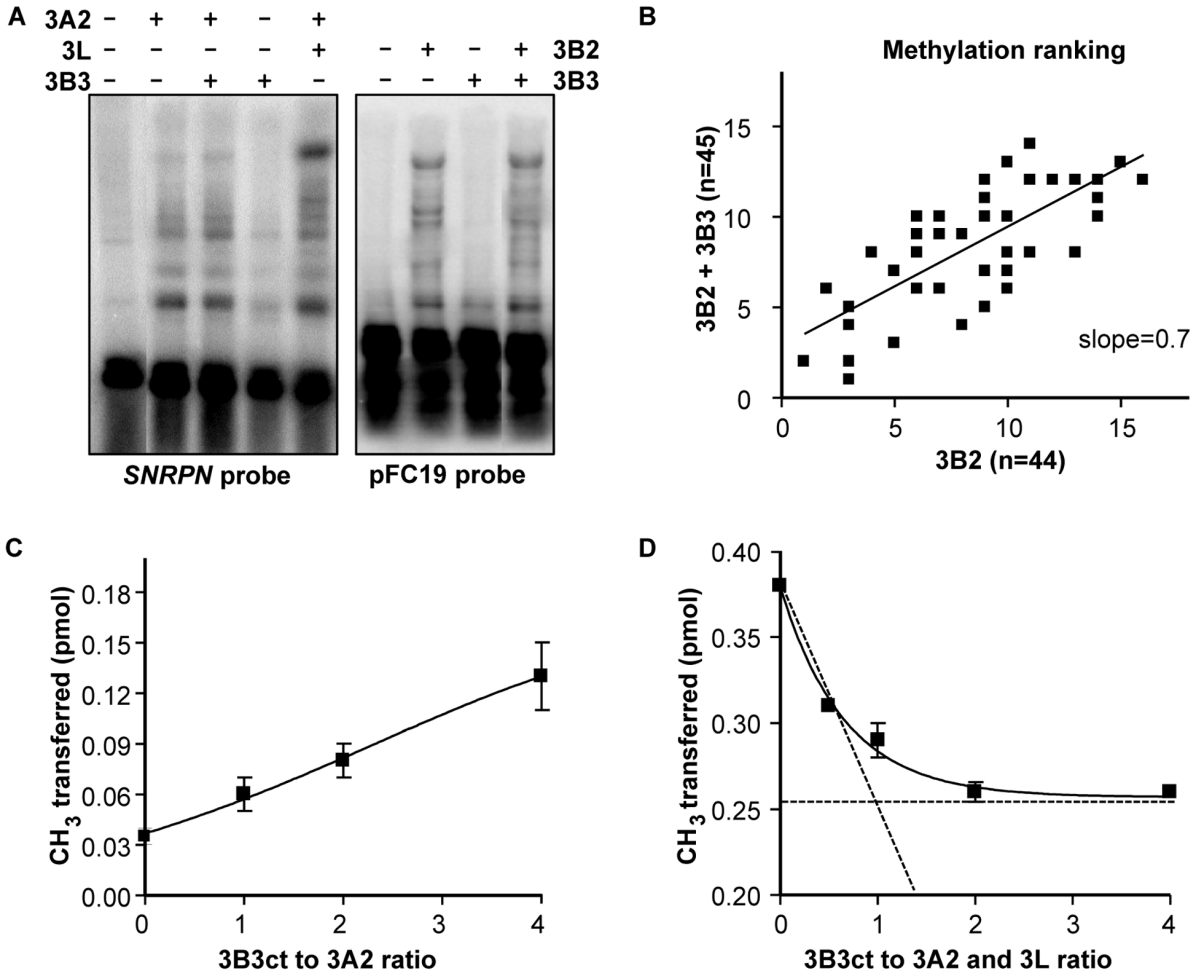
DNMT3B3 modulates DNA methylation activity without affecting DNA methylation patterns. (A) HEK293c18 cells were transfected with the pFC19 target episome and combinations of DNMT3 expression vectors, as indicated. DNA methylation was assessed by Southern blot after digestion of episomal DNA with a methylation-sensitive restriction enzyme. Higher molecular weight bands are indicative of DNA methylation. (B) Bisulfite sequencing was performed on a 500 base pair region of harvested episomal DNA. Co-expression of DNMT3B2 with DNMT3B3 does not lead to a change in DNA methylation patterns as judged by the fact that the ordered ranks of the 48 methylation sites in the region do not shift significantly. (C) Pre-incubation of DNMT3B3 with active DNMT3A2 leads to a stimulation of catalytic activity. The graph displays the result of quantitative *in vitro* activity assays in which the incorporation of labeled methyl groups into DNA was measured at increasing ratios of DNMT3B3ct to DNMT3A2. (D) DNMT3B3 hinders the stimulatory effect of DNMT3L. Pre-incubation of increasing concentrations of DNMT3B3ct to constant amounts of DNMT3A2 and DNMT3L leads to a progressive decline in DNA methylation activity as measured in quantitative *in vitro* assays. Results in panels C and D are from triplicate experiments and shown with average and standard deviations.

In order to determine the effect of DNMT3B3 on DNA methylation, we used a well-characterized episomal assay to measure DNA methyltransferase activity in cell culture [7]. For this, we transfected human HEK293c18 cells with a stably replicating target episome, pFC19, and appropriate pair-wise combinations of DNMT3 isoform expression vectors. After seven to eight days, we recovered episomal DNA by Hirt harvest [31], and assessed DNA methylation first by performing Southern blots with probes to the episome after the DNA was digested with a methylation-sensitive restriction enzyme. Southern blot analysis using three different episomal probes (*SNRPN*, pFC19, or pBR), confirmed that DNMT3B3 by itself is inactive (Figure 2A and Figure S2A). By contrast, co-expression of DNMT3B3 with DNMT3B2 or DNMT3A2, was consistent with active DNA methylation but did not appear to introduce obvious changes in the activity of DNMT3B2 or DNMT3A2 (Figure 2A and Figure S2A).

We next sought to determine whether inclusion of DNMT3B3 in co-complexes could alter the DNA methylation patterns laid by active DNMT3 molecules. To this end, we performed bisulfite DNA methylation sequencing on the top and bottom strand of the pBR region on the pFC19 episome after methylation in HEK293c18 cells (Figure S2B and data not shown). The pBR region, encompassing 500 base pairs and 48 distinct CpG sites, was chosen because it has previously been shown to harbor clear high and low sites for active DNMT3 enzymes [30]. In order to allow comparison of the methylation patterns, we compared the ranks of all CpG sites from highest to lowest based on their frequency of DNA methylation. Consistent with previous data [30], clear high and low efficiency sites were observed, but these DNA methylation patterns remained mostly unchanged upon DNMT3B3 co-expression (Figure 2B, Figure S2B, and data not shown). Likewise, the DNA methylation patterns deposited *in vitro* by purified full-length MBP-DNMT3B2:GST-DNMT3B2 and MBP-DNMT3B2:GST-DNMT3B3 co-complexes on pFC19 episomal DNA were similar to each other (Figure S3). Taken together, our data confirms that DNMT3B3 is associated with active DNA methylation [25] in a manner that does not alter patterns of DNA methylation laid by active DNMT3 molecules.

### DNMT3B3 Modulates the Frequency of *de novo* DNA Methylation in Quantitative *in vitro* Assays

In order to measure potential subtle changes in DNA methylation activity, we performed quantitative *in vitro* DNA methylation activity assays using purified complexes. Consistent with our *in vivo* data, purified DNMT3B3ct by itself was inactive (data not shown). We titrated increasing amounts of MBP-DNMT3B3ct into reactions containing a constant concentration of full-length active DNMT3A2 protein. The proteins were dialyzed in activity buffer overnight to avoid introducing any buffer distortion and were pre-incubated with tritiated SAM for 15 minutes at 37°C before the reactions were initiated by addition of the linear pFC19 DNA substrate. Activity was then measured after 45 minutes. In this assay, addition of DNMT3B3 led to a progressive and reproducible increase in DNA methylation such that DNMT3A2 activity was 3 to 4-fold higher when a 4-fold molar excess of DNMT3B3 was included in the reaction (Figure 2C). Note that the dose response curve did not show evidence of a plateau which suggests that complex formation between DNMT3A2 and DNMT3B3 was inefficient. Nonetheless, this suggests that DNMT3B3 can modestly stimulate the activity of active DNMT3 enzymes provided that co-complexes can be formed.

During development, DNMT3B3 becomes highly expressed at the onset of differentiation at a time when DNMT3L expression is still high before it gradually decreases [24]. We therefore wished to determine if DNMT3B3 could also affect the frequency of DNA methylation in the presence of DNMT3L. For this, purified MBP-DNMT3L, DNMT3A2 and MBP-DNMT3B3ct were pre-incubated in activity buffer such that DNMT3A2 and DNMT3L were kept at constant, equimolar, concentrations and DNMT3B3 was added at increasing concentrations. Addition of DNMT3B3 led to a progressive reduction in DNA methylation activity such that activity in the presence of DNMT3B3 was up to 30% lower than without it (Figure 2D). The maximal reduction was reached for a 1∶1 molar stoichiometry of DNMT3B3 to DNMT3A2:DNMT3L complexes, suggesting that specific co-complexes were formed. Altogether, this suggests that DNMT3B3 exerts subtle, contrasting effects on DNA methylation activity. By itself, it can modestly stimulate *de novo* activity. When added together with DNMT3L, it can counteract the stimulatory activity of DNMT3L and lead to the formation of complexes with intermediate activity.

### DNMT3B4 Inhibits DNMT3 Function

**Figure 3:**
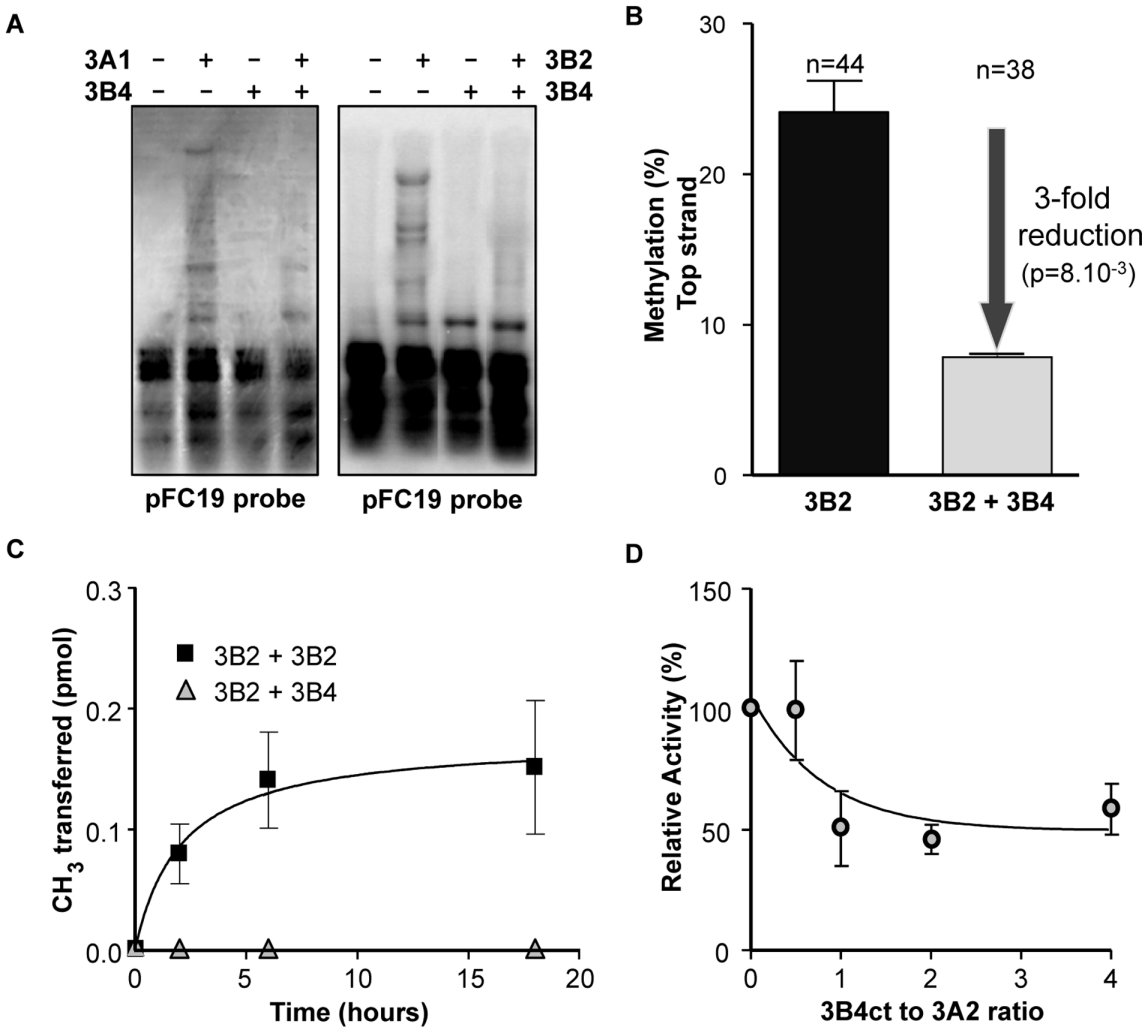
DNMT3B4 inhibits DNMT3 activity. (A) Expression of DNMT3B4 inhibits DNA methylation *in vivo* as measured by Southern blots in transfected HEK293c18 cells. See Figure 2A for further details. (B) *In vivo* methylation mediated by DNMT3B2 on its own (left) or by DNMT3B2 co-expressed with DNMT3B4 (right) was assessed by bisulfite methylation sequencing. Co-expression of DNMT3B4 significantly reduces the overall methylation efficiency over the entire region analyzed. Two independent transfections were analyzed. Bars: standard deviation. (C) Purified full-length co-complexes that include DNMT3B4 are catalytically deficient as measured in *in vitro* time course experiments where the incorporation of labeled methyl groups onto DNA is followed. (D) Pre-incubation of purified DNMT3B4 with DNMT3A2 leads to an inhibition of catalytic activity. The graph depicts results from activity assays that followed the incorporation of labeled methyl groups onto DNA at increasing ratios of DNMT3B4ct to DNMT3A2. Results in panels C and D are from duplicate experiments and shown with average and standard deviations.

The impact of DNMT3B4 on DNA methylation activity was first assessed using our *in vivo* episomal assay. Southern blots consistently showed that co-expression of DNMT3B4 with DNMT3B2, DNMT3A1, or DNMT3A2 resulted in a noticeable reduction in DNA methylation when compared to expression of each active enzyme alone (Figure 3A, Figure S4A, and data not shown). This suggests that DNMT3B4 serves as a broad inhibitor of DNMT3 function. Using bisulfite DNA methylation sequencing on the same region analyzed above, we observed that co-expression of DNMT3B4 with DNMT3B2 led to a significant (p = 0.0082) 3-fold decrease in activity, consistent with DNMT3B4 acting as a dominant-negative inhibitor *of de novo* DNA methylation (Figure 3B). DNA methylation patterns, however, seemed for the most part unchanged (Figure S4B).

We performed *in vitro* activity assays to assess DNA methylation activity of purified DNMT3B2, DNMT3B4, DNMT3B2:DNMT3B2 co-complexes, as well as DNMT3B2:DNMT3B4 and DNMT3B4:DNMT3A co-complexes. Consistent with our *in vivo* data, we observed that DNMT3B4ct by itself was inactive while DNMT3B2ct and DNMT3B2:DNMT3B2 co-complexes (both full-length and C-terminal) were active (data not shown). Purified DNMT3B2:DNMT3B4 full-length and C-terminal co-complexes were inactive, consistent with DNMT3B4 strongly inhibiting DNMT3B function through complex formation (Figure 3C and data not shown). Additionally, we show that pre-incubation of MBP-DNMT3B4ct with active full-length DNMT3A2 also hindered DNMT3A function *in vitro*, consistent with *in vivo* data (Figure 3D). Taken together, our data suggests that DNMT3B4 is a dominant-negative inhibitor of active DNMT3 function.

### DNMT3B3 Weakens, While DNMT3B4 Strongly Inhibits, DNA Binding by Active DNMT3B Molecules

**Figure 4:**
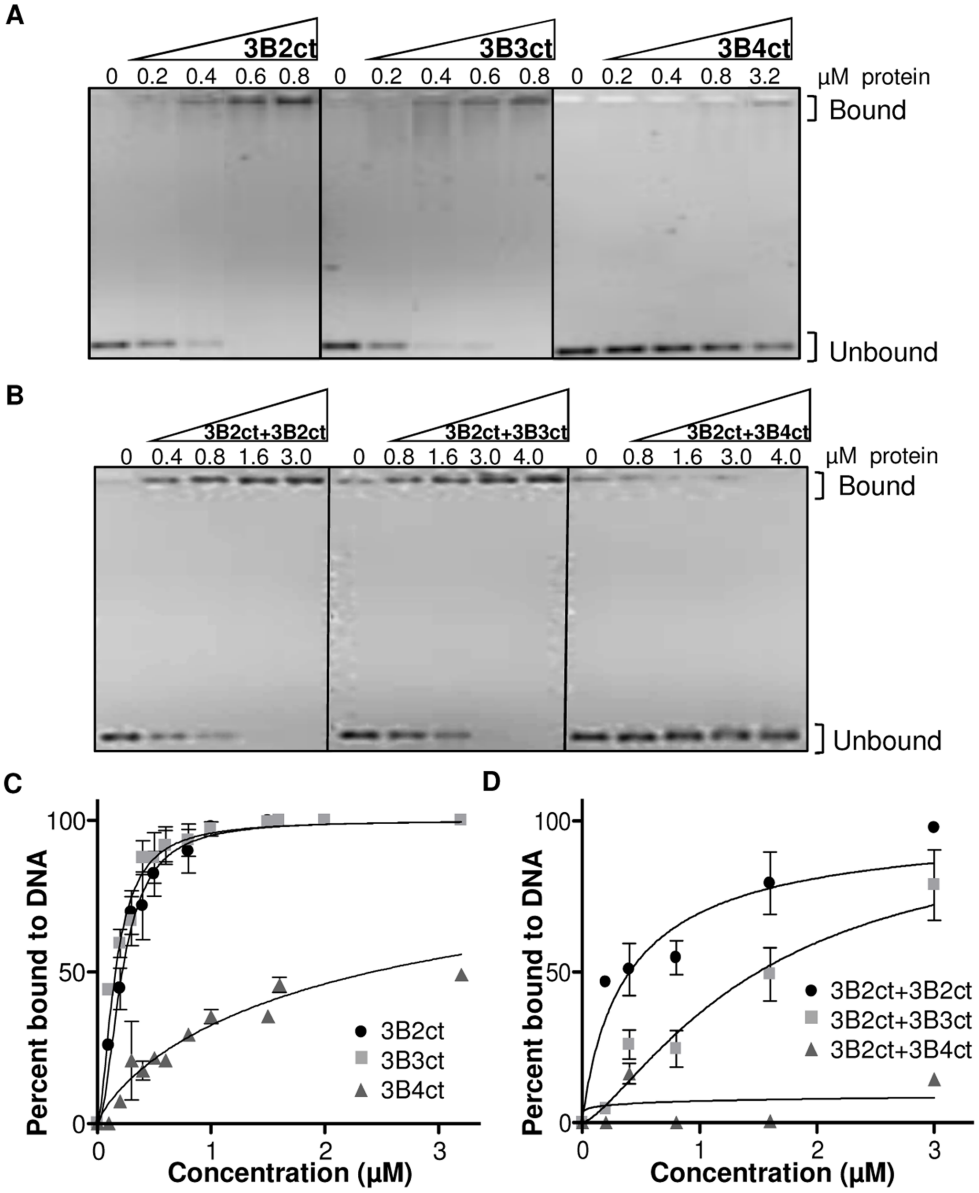
DNMT3B3 weakens, while DNMT3B4 strongly inhibits, DNA binding by DNMT3B2. (A) Binding to a 420 base pair DNA fragment was measured by EMSAs for increasing concentrations of C-terminal DNMT3B2, DNMT3B3 and DNMT3B4 proteins. Bound and unbound DNA species are indicated. (B) DNA binding was also measured for C-terminal DNMT3B2:DNMT3B2, DNMT3B2:DNMT3B3, and DNMT3B2:DNMT3B4 co-complexes at increasing protein concentrations. (C and D) Graphical representation of EMSAs from A and B, respectively, performed at least in triplicate. The percent of protein bound to DNA was calculated by band quantification using ImageQuant. Points: mean, bars: standard error. Results were fit using nonlinear regression, saturation-binding (one-site-specific binding with Hill slope). Kd values: 3B2ct = 0.22 µM, 3B3ct = 0.16 µM, 3B4ct = 2.42 µM, 3B2ct+3B2ct = 0.4 µM, 3B2ct+3B3ct = 1.5 µM, 3B2ct+3B4ct = N/A.

To better understand the mechanism(s) by which DNMT3B3 and DNMT3B4 exert their effects on active DNMT3 molecules, we investigated the DNA binding properties of DNMT3B2, DNMT3B3, and DNMT3B4 either by themselves or as co-complexes. For this, we performed electromobility shift assays (EMSAs) with purified C-terminal DNMT3B2, DNMT3B3, and DNMT3B4 isoforms; and C-terminal and full-length DNMT3B2:DNMT3B2, DNMT3B2:DNMT3B3, and DNMT3B2:DNMT3B4 co-complexes (Figure 4A, 4B and Figure S5). By itself, C-terminal DNMT3B3 bound to a test DNA fragment with similar affinity as C-terminal DNMT3B2; however, C-terminal DNMT3B4 bound to DNA significantly less than C-terminal DNMT3B2, as shown by the fact that its Kd for DNA was 10-fold higher than the one measured for DNMT3B2 (Figure 4C). In similar EMSA experiments performed using both C-terminal and full-length DNMT3B co-complexes, we noticed that DNMT3B2:DNMT3B2 co-complexes consistently bound DNA with a slightly higher affinity than DNMT3B2:DNMT3B3 co-complexes (Figure 4B, 4D and Figure S5), suggesting that DNMTB3 may weaken DNA binding by DNMT3B2. In all cases, DNMT3B2:DNMT3B4 co-complexes bound to DNA much less than DNMT3B2 homo-complexes (Figure 4B, 4D and Figure S5). The poor ability of DNMT3B4 and DNMT3B4-containing complexes to bind to DNA suggests that DNMT3B4 might exert its inhibitory effects by sequestering active DNMT3 molecules away from their DNA substrate. Binding in the presence of DNMT3B3, by contrast, is only slightly reduced. In all cases, we noted that the full-length DNMT3B co-complexes bound to DNA with a higher affinity than C-terminal co-complexes, which suggests that the DNMT3B N-terminus is involved in DNA binding, as reported earlier [32].

### DNMT3B Isoforms Drive Distinct DNA and H3K9me3 Staining

**Figure 5:**
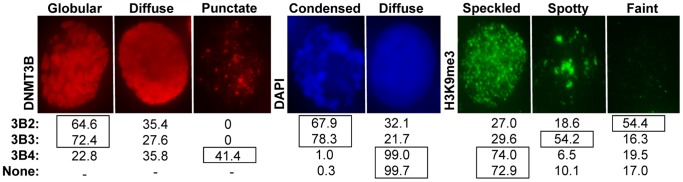
DNMT3B isoforms drive unique and distinct localization, DNA staining, and H3K9me3 patterns in human cells. FLAG-tagged DNMT3B2, DNMT3B3, and DNMT3B4 were transiently transfected into human HEK293 cells. 24 to 36 hours later, cells were fixed and stained with anti-FLAG (Sigma) and anti-H3K9me3 (Abcam) antibodies. Transfections were repeated at least in triplicate and cells were photographed with a fluorescence microscope. Representative DNMT3B (Globular, Diffuse, or Speckled: red), DNA (Condensed or Diffuse: blue), and H3K9me3 (Speckled, Spotty, or Faint: green) staining are depicted. Percentages of each type of staining versus DNMT3B isoform expressed are indicated below each image. The total number of independent cells analyzed: DNMT3B2, n = 237; DNMT3B3, n = 203; DNMT3B4, n = 215; none, n = 356.

In order to gain further insights into how expression of DNMT3B3 and DNMT3B4 might exert their functions *in vivo*, we transiently overexpressed FLAG-tagged DNMT3B2, DNMT3B3, and DNMT3B4 in HEK293 cells, and compared their localization patterns using immunocytochemistry. Three different DNMT3B localization patterns were observed: globular, diffuse, and punctate (Figure 5). The majority of DNMT3B2 and DNMT3B3-expressing cells displayed a globular expression pattern, which was the least likely expression pattern for DNMT3B4-overexpressing cells (Figure 5). DNMT3B4, in contrast, displayed a punctate distribution pattern, which was not observed in any of the DNMT3B2 or DNMT3B3-expressing cells (Figure 5). Similar localization patterns were also observed in mouse NIH3T3 cells (Figure S6).

Interestingly, DNA organization, as observed by DAPI staining, also varied depending on the specific DNMT3B isoform being expressed (Figure 5). The majority of DNMT3B2 or DNMT3B3-overexpressing cells displayed a condensed DNA staining pattern. In most cases, this condensed staining pattern was associated with the DNMT3B2 or DNMT3B3 globular localization pattern, suggesting that expression of DNMT3B2 or DNMT3B3 leads to the formation of condensed, DAPI-bright, regions, an observation consistent with the fact that DNMT3B associates with components of the condensin complex [33]. DNMT3B4-expressing cells, on the other hand, mostly displayed a diffuse DAPI staining pattern no matter the type of DNMT3B4 expression pattern. The DNA staining of DNMT3B4-expressing cells was similar to the DNA staining observed in untransfected cells, suggesting that DNMT3B4 expression does not alter DNA organization and/or compaction. Expression of human DNMT3B4 in mouse NIH3T3 cells, similarly, resulted in DNA staining typical of untransfected cells (Figure S6).

Given that DNMT3B is targeted to compacted, histone 3 lysine 9 tri-methylation (H3K9me3)-rich, pericentric heterochromatic regions [34],[35], we also analyzed whether the various DNMT3B isoforms studied here caused any perturbation in the H3K9me3 staining pattern. In untransfected HEK293 cells, the H3K9me3 distribution was speckled in the majority of cases (Figure 5). In a minority of cells, the H3K9me3 pattern was pronounced and spotty, or alternatively, faint (Figure 5). Interestingly, expression of DNMT3B2 and DNMT3B3 isoforms resulted in distinct alterations of the H3K9me3 staining pattern. DNMT3B2 expression shifted the H3K9me3 staining to a mostly faint pattern while DNMT3B3 expression shifted the distribution to a mostly spotty pattern. Most DNMT3B4-expressing cells displayed a speckled H3K9me3 staining corresponding to the typical H3K9me3 staining of untransfected cells (Figure 5). A similar reduction in H3K9me3 staining was observed when human DNMT3B2 was expressed in mouse NIH3T3 cells (Figure S6). An increase in spottiness upon DNMT3B3 expression could not be observed upon DNMT3B3 expression in NIH3T3 cells (Figure S6); however, this is likely due to the large chromocenters characteristic of mouse NIH3T3 cells [36]. Like in HEK293 cells, DNMT3B4-expressing NIH3T3 cells displayed characteristics resembling most closely those of untransfected cells.

Since the majority of cells expressing DMT3B2 displayed a faint H3K9me3 staining, we hypothesized that expression of DNMT3B2 might be causing global H3K9me3 levels to be reduced. In order to test this idea, we transfected HEK293 cells with increasing amounts of DNMT3B2-expressing vector and used quantitative western blotting to measure H3K9me3 levels with a modification-specific antibody. Gradual overexpression of DNMT3B2 as a function of vector amounts was verified via western blot with an anti-FLAG antibody (data not shown). Equal amounts of histone-extracted samples were then loaded on an SDS-PAGE gel and processed for western blot with an anti-H3K9me3 antibody. No consistent decrease in global H3K9me3 levels upon expression of DNMT3B2 could be demonstrated despite multiple attempts (data not shown), suggesting that DNMT3B2 expression might be causing a re-distribution of H3K9me3 rather than a global reduction. Further experiments are needed to confirm this hypothesis.

## Discussion

Using DNMT3L as a paradigm for the regulation of active DNMT3 enzymes by inactive DNMT3 variants, we set out to determine if inactive DNMT3B splice variants could bind to and modulate the activity of catalytically competent DNMT3 members. Our results reveal that the DNMT3B3 and DNMT3B4 variants follow this paradigm, albeit result in distinct consequences on activity and involve distinct types of interactions.

Like previous studies with DNMT3L, we show that inactive DNMT3B3 and DNMT3B4 proteins bind to active DNMT3A and/or DNMT3B molecules *in vivo* and *in vitro*, although the types of complexes formed appear distinct from those formed by DNMT3L. As observed for DNMT3A [1],[12],[37], active DNMT3B isoforms both self-interact and interact with each other, which is consistent with several prior studies [10],[32],[38]. Unlike DNMT3L, however, DNMT3B3 and DNMT3B4 formed large soluble homo-complexes, an observation reported independently for DNMT3B3 [32]. This is surprising, particularly in the case of DNMT3B4, given the resemblance of the C-terminus of these isoforms to DNMT3L (Figure S1A), a protein that exists mostly as monomers or dimers in solution [11],[12]. This suggests that there are uncharacterized DNMT3B3 and DNMT3B4 self-interaction regions within the C-terminus of these isoforms. In addition, the reorganization of large active DNMT3A or 3B complexes into smaller heterodimers observed upon DNMT3L interaction [11],[12] does not seem to occur upon DNMT3B3 or DNMT3B4 interaction. Gel filtration chromatography instead revealed that purified DNMT3B2:DNMT3B2, DNMT3B2:DNMT3B3, and DNMT3B2:DNMT3B4 co-complexes still exist as large soluble aggregates. Despite this, western blots suggested that these co-complexes are of defined stoichiometries. DNMT3B2 interacts with DNMT3B3 in a 1∶1 molar ratio, while DNMT3B2 and DNMT3B4 appear to interact with a 1∶2 ratio. It will be interesting to determine how subtle differences in the domain structures of DNMT3B3 and DNMT3B4 (see Figure S1A) account for the differences in stoichiometric ratios once isoform-specific structural information becomes available. The functional relevance of the DNMT3B2:DNMT3B4 stoichiometric ratio is unclear, although it might ensure that overexpression of DNMT3B4 is necessary to reveal the effect of DNMT3B4. It should be noted that the ability of DNMT3B3 or DNMT3B4 to interact with active DNMT3A or DNMT3B isoforms is much reduced compared to DNMT3L. Unlike DNMT3L, simple pre-incubation of various isoforms in activity buffer was mostly insufficient to allow formation of mixed complexes (data not shown). Instead, mixed co-complexes could be most readily recovered if the isoforms were either co-expressed or co-purified together directly from cells. Taken together, our data demonstrates that inactive DNMT3B variants can participate in mixed co-complexes with active DNMT3 enzymes, but that their mode of interactions are distinct from those described previously for DNMT3L.

**Figure 6:**
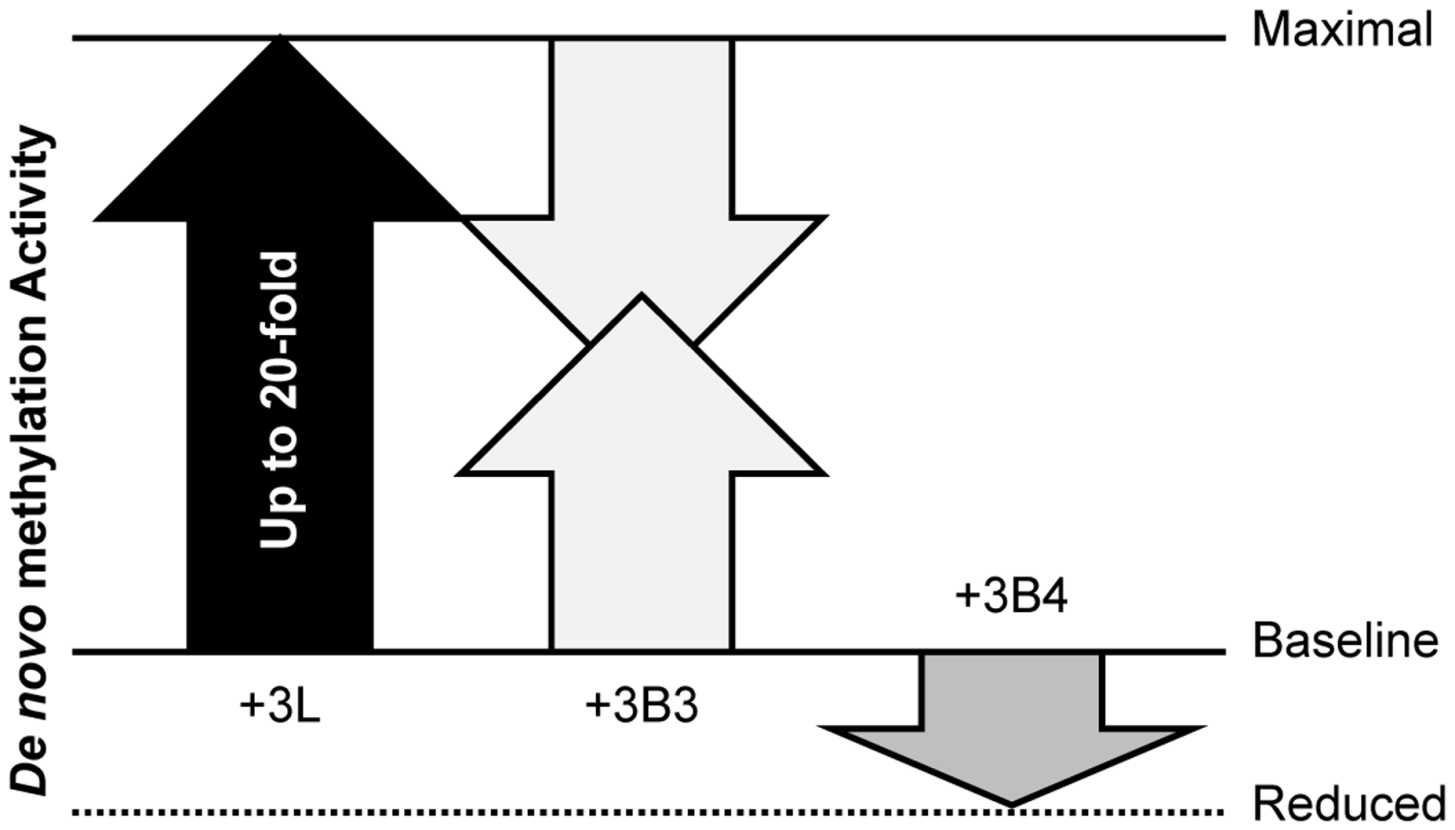
Inactive DNMT3 variants modulate DNA methylation activity. DNMT3L, the prototypic inactive DNMT3 variant stimulates *de novo* methylation activity up to 20-fold from a baseline level up to maximal methylation levels [12]. Stimulation is graphically depicted by a vertical arrow. DNMT3B3, by contrast, can both hinder the stimulatory effect of DNMT3L (shown by a downward pointing arrow) and stimulate the activity of active DNMT3s from their baseline levels (shown by an upward pointing arrow). DNMT3B4, by contrast, inhibits DNA methylation 3-fold lower than baseline level. Altogether, inactive DNMT3 variants can modulate *de novo* methylation activity over a 60-fold range.

While DNMT3L, DNMT3B3, and DNMT3B4 all bind to active DNMT3 members, they have drastically different effects on DNA methylation efficiency. The DNMT3L stimulatory factor can enhance *de novo* DNA methylation up to 20-fold in reconstituted biochemical assays [10],[12]. In the case of DNMT3B4, interaction results in a 3-fold reduction of activity, consistent with several reports linking DNMT3B4 expression to DNA hypomethylation [20],[29]. The DNMT3B3 splice variant, by contrast, stimulated *de novo* DNA methylation, but did so modestly, leading to a 3 to 4-fold increase of activity (Figure 2C). This observation is consistent with evidence that the expression of DNMT3B3 is associated with active DNA methylation [25] and with a previous report suggesting that DNMT3B3 may act as a weak stimulator of DNA methylation [32]. Unlike DNMT3L, which exerts its maximal stimulatory effect at a 1∶1 molar ratio, we observed that excess concentrations of DNMT3B3 over DNMT3A2 were required for stimulation. This probably reflects the fact that DNMT3B3:DNMT3A2 co-complexes form only inefficiently under our conditions. If correct, this suggests that the true ability of DNMT3B3 to stimulate *de novo* methylation may have been under-estimated in our assays. Interestingly, incubation of DNMT3B3 together with DNMT3A2 in the presence of equimolar DNMT3L concentrations led to a progressive and reproducible reduction of DNA methylation efficiency such that activity was 30% reduced by DNMT3B3. Unlike what we observed for bi-molecular complexes, the maximal effect of DNMT3B3 was observed for a 1∶1 stoichiometry of DNMT3B3 to DNMT3A2 and DNMT3L (Figure 2D). This striking difference in dose response between bi- and tri-molecular complexes suggests that DNMT3L may facilitate the interaction between DNMT3B3 and DNMT3A2. Whether tri-molecular DNMT3B3:DNMT3A2:DNMT3L or bi-molecular DNMT3B3:DNMT3A2 complexes ultimately result from the interaction of these three proteins remains to be determined. It should be noted that a recent study [32] reported that DNMT3B3 caused a ∼20% increase in DNA methylation in the context of tri-molecular DNMT3B3:DNMT3A2:DNMT3L complexes, contrasting with the ∼30% decrease reported here. The difference between these studies could be due to differences in buffer conditions (high EDTA in [32] compared to a more physiological Mg-containing buffer here) or to the fact that our studies were limited to the C-terminal domain of DNMT3B3. Altogether, our study suggests that DNMT3B3 is capable of counteracting the stimulatory function of DNMT3L. In the absence of DNMT3L, however, DNMT3B3 appears to stimulate the activity of DNMT3 enzymes although this property may be limited by the difficulty of forming bi-molecular complexes. Such contrasting behavior may enable the transition from a maximal *de novo* methylation activity fueled by DNMT3L in early development to an intermediate level once DNMT3B3 becomes expressed. Once DNMT3L expression is down-regulated, DNMT3B3 expression, which is common in most somatic cells, could maintain *de novo* DNA methylation slightly above a baseline level in the absence of DNMT3L. Such a hypothesis is consistent with the dynamics of expression of the various isoforms involved [24], and the dynamics of DNA methylation activity through development (Figure S7). Altogether, our results show that inactive DNMT3 variants including DNMT3L and DNMT3B splice variants, can have a profound impact on *de novo* DNA methylation, modulating the efficiency of active DNMT3A or DNMT3B enzymes over at least a 60-fold range (Figure 6).

While all inactive DNMT3 variants studied so far appear to exert their effects on *de novo* DNA methylation through the formation of co-complexes with active DNMT3 molecules, the consequences of these interactions are distinct. DNMT3L operates by promoting the rearrangement of large DNMT3A2 complexes into defined hetero-dimeric sub-complexes with increased catalytic potential, higher affinity for SAM, and higher processivity [11],[12],[13]. DNMT3L, however, does not seem to affect DNA binding by DNMT3A [12]. DNMT3B4, by contrast, appears to cause a sharp reduction in DNA binding affinity in its co-complexes. We suggest that this reduction is the cause of the broad inhibitory effects of this isoform on DNA methylation. This is in contrast to the hypothesis that DNMT3B4 might function by outcompeting active DNMT3B isoforms for targeting to DNA regions, resulting in DNA hypomethylation [20]. Indeed this model is not compatible with our evidence that DNMT3B4 binds to DNA only weakly, suggesting instead that DNMT3B4 functions by sequestering active DNMT3 molecules away from DNA. Unlike DNMT3B4, the DNMT3B3 isoform is still capable of strong DNA binding albeit DNMT3B3:DNMT3B2 co-complexes show slightly reduced binding, in agreement with an independent report [32]. We suggest that the slightly weakened DNA binding might result in faster turnover of co-complexes, thereby explaining the ability of DNMT3B3 to modestly stimulate DNA methylation. This interpretation is consistent with analyses indicating that the rate-limiting step for catalysis by the model HhaI cytosine methyltransferase corresponds to the final dissociation step from the reaction’s products [39]. Furthermore, we note that a DNMT3B1 ICF syndrome mutation, R823G, was recently reported to show a profound DNA methylation defect caused by DNA dissociation defect [10].

In addition to affecting intrinsic catalytic properties, it is possible that DNMT3B variants also modulate *de novo* DNA methylation indirectly through effects on chromatin condensation and modification. Immunocytochemistry experiments suggest that expression of DNMT3B2 or DNMT3B3, but not DNMT3B4, drive chromatin condensation. This is consistent with the fact that overexpression of Dnmt3a in fly causes irregular chromosome condensation, DNA hypermethylation, and elevated levels of both di-and tri-methyl H3K9 in pericentromeric regions [40]. Likewise, it is consistent with the fact that DNMT3B has been shown to interact with components of the condensin complex [33]. Interestingly, our immunofluorescence experiments also revealed that expression of DNMT3B2, DNMT3B3, and DNMT3B4 had different effects on H3K9me3 staining. While DNMT3B4-expressing and untransfected cells mainly displayed a speckled H3K9me3 staining pattern, DNMT3B2-expressing cells mainly showed a faint H3K9me3 staining. By contrast, DNMT3B3-expressing cells mainly displayed localized “spotty” increases in H3K9me3 staining. The significance of these results remains to be clarified, but fall into an accumulating body of data showing that H3K9me3, DNA methylation, and DNMT3B are tightly linked [41],[42],[43]. Since DNMT3B3 expression appears to favor DNA condensation and the formation of H3K9me3-dense regions, DNMT3B3 might favor the redirection of *de novo* DNA methylation towards pericentric H3K9me3-rich heterochromatic regions. DNMT3B4, on the other hand, does not appear to be associated with H3K9me3 and its diffuse localization pattern is consistent with poor binding to DNA. DNMT3B4 expression could thus further drive a hypomethylation phenotype by severing the connection between active DNMT3A and DNMT3B from their normal targets both at the DNA and chromatin levels. This notion is consistent with the observation of DNA hypomethylation at pericentromeric satellite sequences upon overexpression of DNMT3B4 in human hepatocarcinoma [20].

In summary, our work contributes new insights into the regulation of *de novo* DNA methylation by inactive DNMT3B isoforms in human cells. Most importantly, our work provides clear evidence that inactive DNMT3B3 and DNMT3B4 splice variants, like DNMT3L, interact with active DNMT3 family members and modulate DNMT3 function upon complex formation, providing a general mechanism for the regulation of the activity of DNMT3 family members. Likewise, our work illustrates how overexpression of inactive DNMT3B variants may lead to the generation of aberrant DNA methylation patterns in diseases such as cancer. Finally, our work indicates that while most studies have naturally focused on understanding the function of active DNMT3A or DNMT3B molecules, the potential regulatory role of inactive DNMT3 variants should not be ignored, especially in light of the fact that over thirty, mostly inactive, isoforms are expressed from the *DNMT3B* locus alone.

## Supporting Information

Figure S1 **DNMT3 variants and protein purity.** (A) Schematic of the structure of DNMT3 variants with major protein domains indicated. (B and C) Aliquots (∼1 µg) of purified DNMT3 co-complexes and proteins run on 8% SDS-polyacrylamide gels stained with Coomassie Brilliant Blue.
(TIF)

Figure S2 **DNMT3B3 modulates DNA methylation activity without affecting DNA methylation patterns.** (A) HEK293c18 cells were transfected with the pFC19 target episome and combinations of DNMT3 expression vectors, as indicated. DNA methylation was assessed by Southern blot with the pBR probe after digestion of episomal DNA with a methylation-sensitive restriction enzyme. Higher molecular weight bands are indicative of DNA methylation. (B) *In vivo* methylation mediated by DNMT3B2 on its own (top) or by DNMT3B2 in the presence of DNMT3B3 (bottom) was assessed by bisulfite methylation sequencing (the pBR 500 base pair region containing 48 CpG sites was chosen). Two independent transfections were analyzed and combined. Closed symbols indicate methylation, open symbols indicate no methylation. The patterns of methylation do not appear to have shifted as high methylation sites and low methylation sites largely remain the same.
(TIF)

Figure S3 **DNMT3B3 does not alter DNA methylation patterns** ***in vitro*** **.** Activity assays were performed with purified full-length DNMT3B2:DNMT3B2 and DNMT3B2:DNMT3B3 co-complexes on pFC19 DNA overnight. Bisulfite sequencing was performed on a 500 base pair region of the episome revealing that DNMT3B3 does not lead to a significant change in DNA methylation patterns as judged by the lack of significant shift in the rankings of the 48 methylation sites analyzed here.
(TIF)

Figure S4 **DNMT3B4 inhibits DNA methylation activity of DNMT3B2 but does not alter DNA methylation patterns** ***in vivo.*** (A) HEK293c18 cells were transfected with the pFC19 target episome and combinations of DNMT3 expression vectors, as indicated. DNA methylation was assessed by Southern blot with the pBR probe after digestion of episomal DNA with a methylation-sensitive restriction enzyme. Higher molecular weight bands are indicative of DNA methylation. (B) Patterns of DNA methylation mediated by DNMT3B2 (top) or DNMT3B2 in the presence of DNMT3B4 (bottom) were assessed by bisulfite methylation sequencing. Two independent transfections were analyzed and combined. Closed symbols indicate methylation, open symbols indicate no methylation. While an overall reduction of DNA methylation is clearly observed, the patterns do not appear to have shifted.
(TIF)

Figure S5 **DNMT3B3 and DNMT3B4 hinder DNA binding by DNMT3B2.** Representative EMSA gels for full-length DNMT3B2:DNMT3B2, DNMT3B2:DNMT3B3, and DNMT3B2:DNMT3B4 complexes at increasing protein concentrations are shown. A 420 base pair DNA fragment (0.1 µM) was used as a target.
(TIF)

Figure S6 **DNMT3B isoforms drive unique and distinct localization, DNA staining, and H3K9me3 patterns in mouse cells.** Human FLAG-tagged DNMT3B2, DNMT3B3, or DNMT3B4 were transiently transfected into mouse NIH3T3 cells and immunofluorescence experiments performed with anti-FLAG and anti-H3K9me3 antibodies. See Figure 5 for further details. The total number of independent cells analyzed: DNMT3B2, n = 122; DNMT3B3, n = 66; DNMT3B4, n = 58; none, n = 486.
(TIF)

Figure S7 **DNMT3 variant expression and** ***de novo*** **DNA methylation during mammalian development.** DNMT3A2, DNMT3L, and DNMT3B1 are highly expressed during early development, taking part in establishing global DNA methylation patterns. Upon differentiation, DNMT3B3 becomes highly expressed while DNMT3B1 expression is abruptly shut down. During development, expression of DNMT3A and DNMT3B gradually shifts to DNMT3A1 and DNMT3B2, respectively, while DNMT3L expression is gradually reduced. DNMT3A1, DNMT3B2, and DNMT3B3 take part in completing global DNA methylation patterns. DNMT3B3 is the major splice variant in somatic cells [23] and likely plays a role in directing DNA methylation towards condensed H3K9me3-rich pericentric repeats. Adult tissues generally express low levels of DNMT3 variants and display tissue specific expression of DNMT3B4. In Hepatocellular Carcinomas (HCC) DNMT3B4 becomes highly expressed, and such overexpression is associated with global loss of DNA methylation at pericentric repeats [21].
(TIF)
