# Supplementary figures and images for: Inactive DNMT3B Splice Variants Modulate De Novo DNA Methylation
Source: PLoS One. 2013 Jul 19;8(7):e69486. doi: 10.1371/journal.pone.0069486 (PMC3716610; doi:10.1371/journal.pone.0069486)

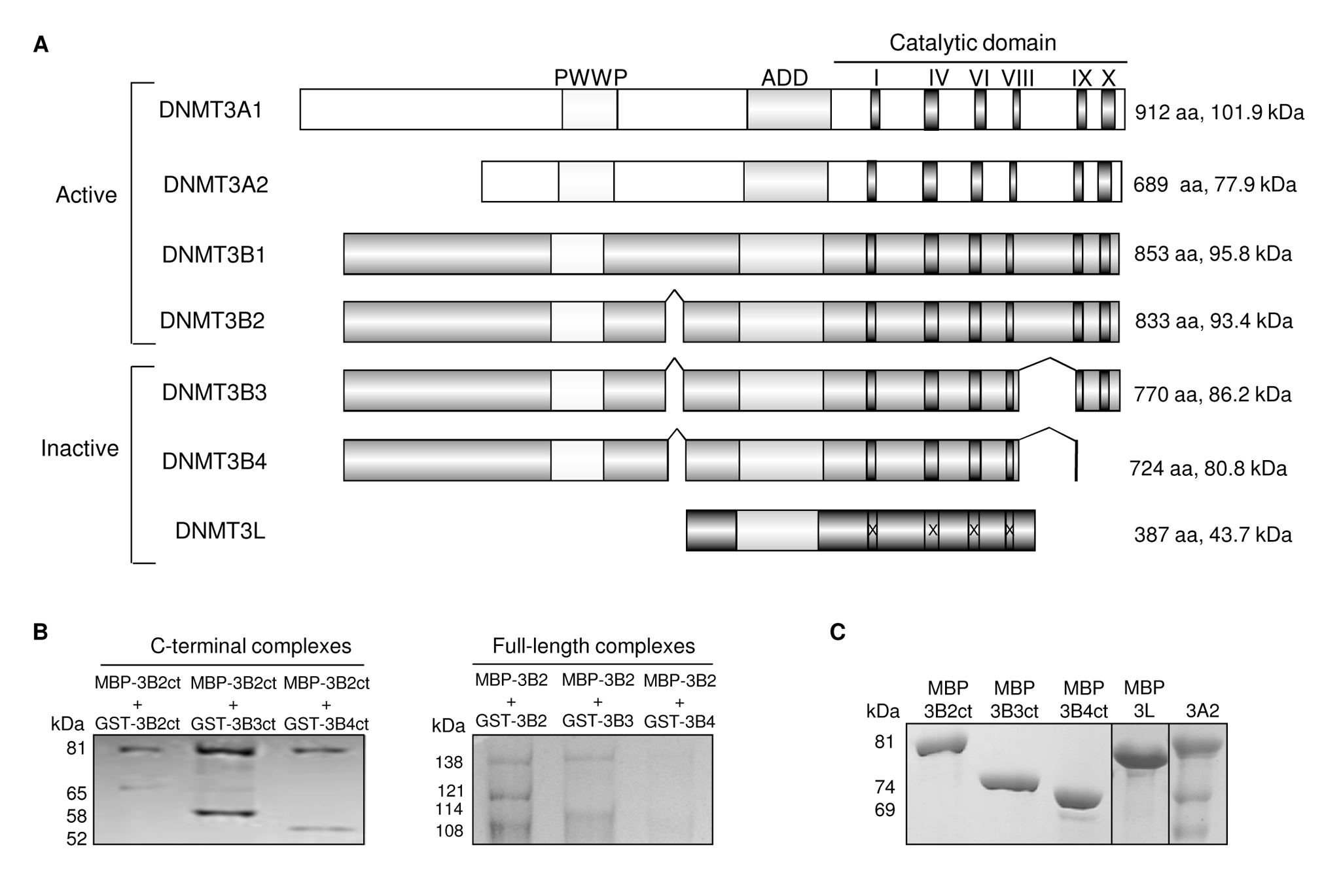

Supplement: Figure S1 — DNMT3 variants and protein purity. (A) Schematic of the structure of DNMT3 variants with major protein domains indicated. (B and C) Aliquots (∼1 µg) of purified DNMT3 co-complexes and proteins run on 8% SDS-polyacrylamide gels stained with Coomassie Brilliant Blue. (TIF) [file pone.0069486.s001.tif]

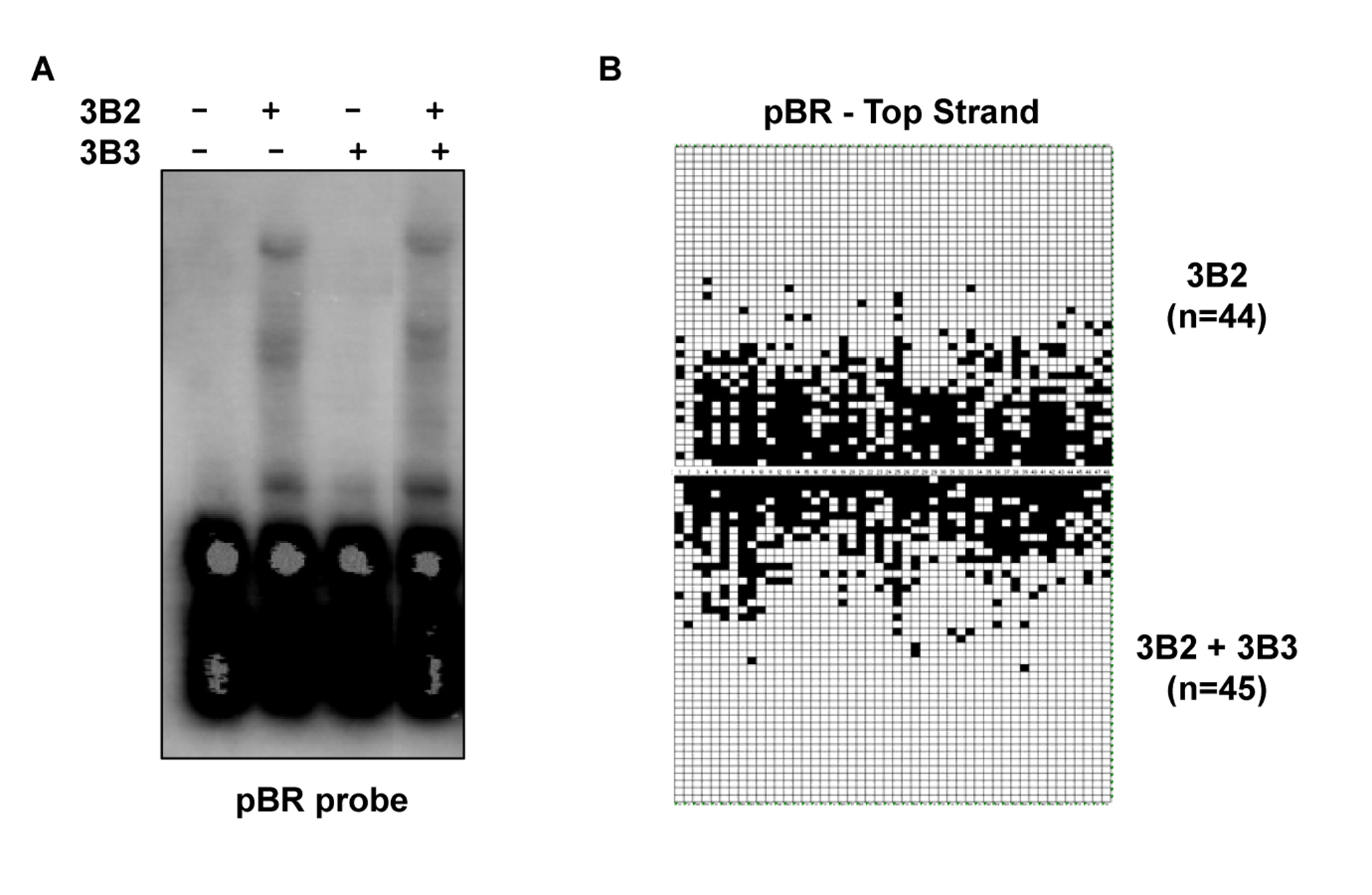

Supplement: Figure S2 — DNMT3B3 modulates DNA methylation activity without affecting DNA methylation patterns. (A) HEK293c18 cells were transfected with the pFC19 target episome and combinations of DNMT3 expression vectors, as indicated. DNA methylation was assessed by Southern blot with the pBR probe after digestion of episomal DNA with a methylation-sensitive restriction enzyme. Higher molecular weight bands are indicative of DNA methylation. (B) In vivo methylation mediated by DNMT3B2 on its own (top) or by DNMT3B2 in the presence of DNMT3B3 (bottom) was assessed by bisulfite methylation sequencing (the pBR 500 base pair region containing 48 CpG sites was chosen). Two independent transfections were analyzed and combined. Closed symbols indicate methylation, open symbols indicate no methylation. The patterns of methylation do not appear to have shifted as high methylation sites and low methylation sites largely remain the same. (TIF) [file pone.0069486.s002.tif]

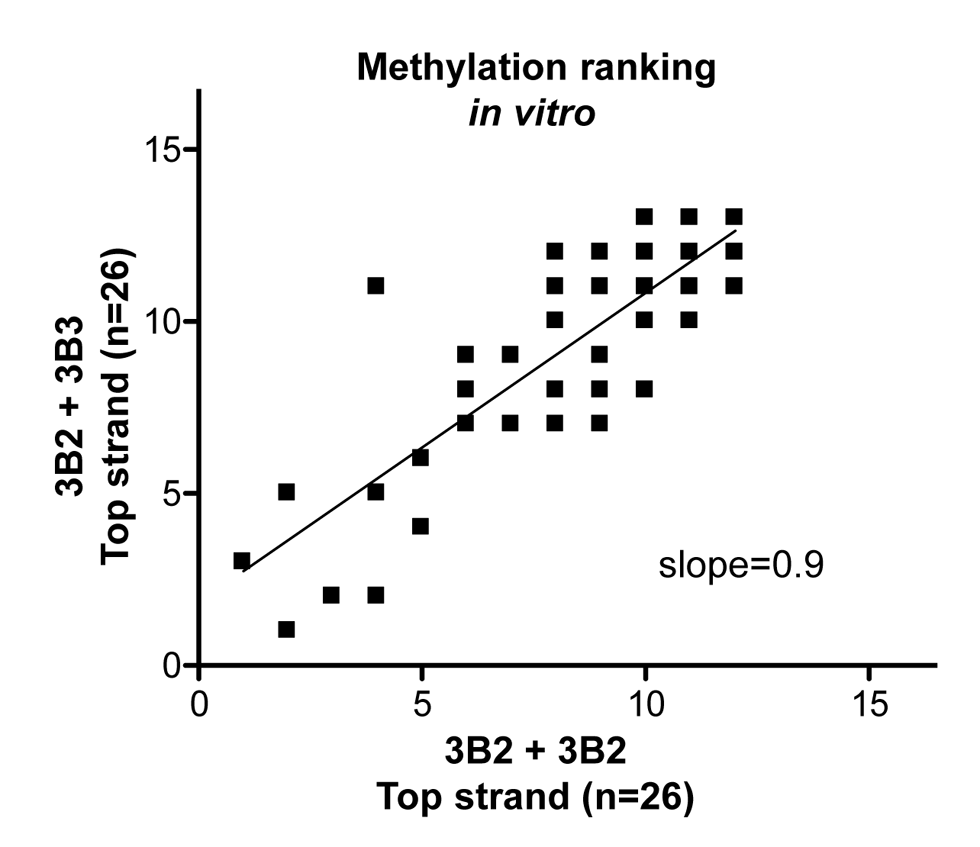

Supplement: Figure S3 — DNMT3B3 does not alter DNA methylation patterns in vitro . Activity assays were performed with purified full-length DNMT3B2:DNMT3B2 and DNMT3B2:DNMT3B3 co-complexes on pFC19 DNA overnight. Bisulfite sequencing was performed on a 500 base pair region of the episome revealing that DNMT3B3 does not lead to a significant change in DNA methylation patterns as judged by the lack of significant shift in the rankings of the 48 methylation sites analyzed here. (TIF) [file pone.0069486.s003.tif]

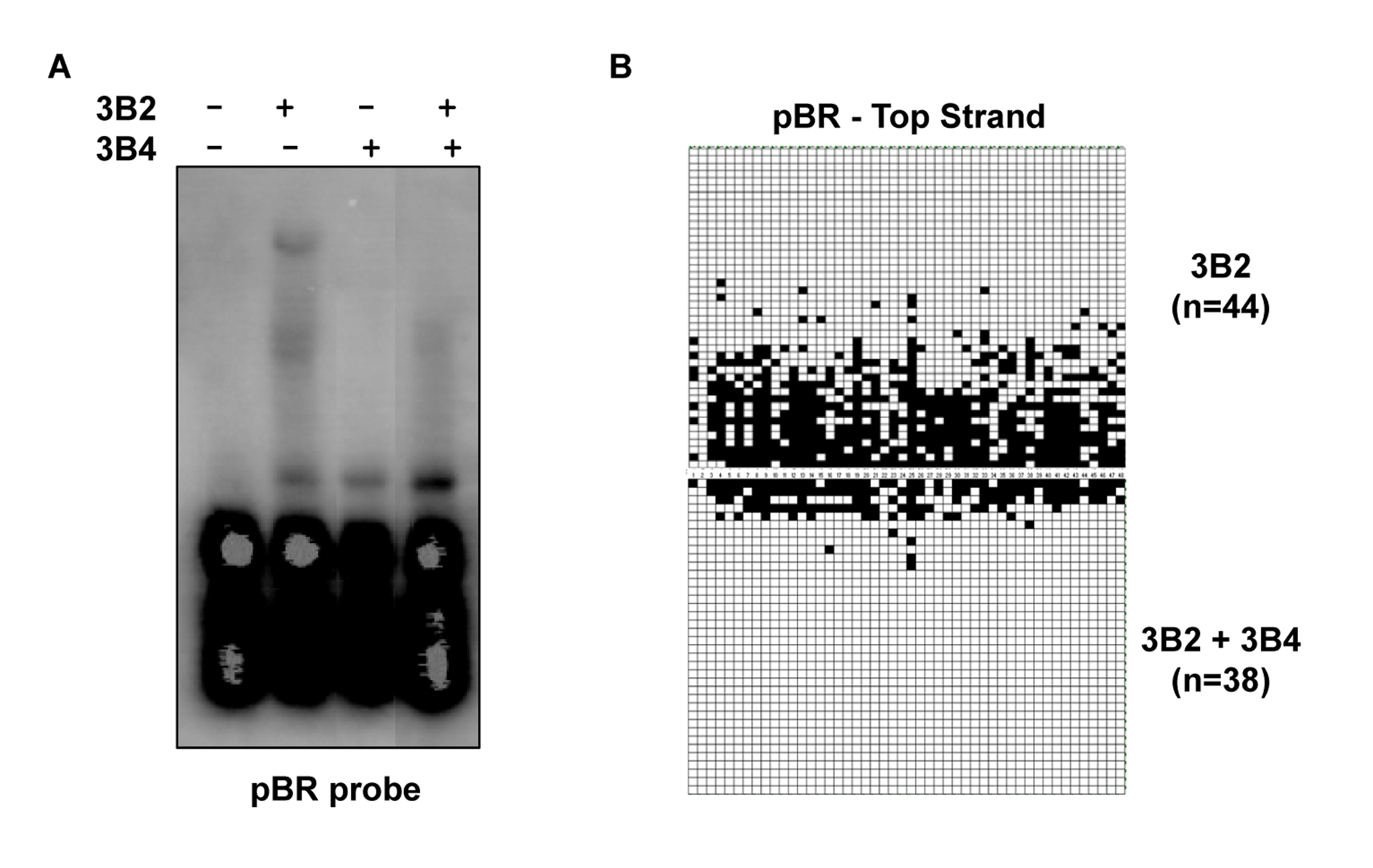

Supplement: Figure S4 — DNMT3B4 inhibits DNA methylation activity of DNMT3B2 but does not alter DNA methylation patterns in vivo. (A) HEK293c18 cells were transfected with the pFC19 target episome and combinations of DNMT3 expression vectors, as indicated. DNA methylation was assessed by Southern blot with the pBR probe after digestion of episomal DNA with a methylation-sensitive restriction enzyme. Higher molecular weight bands are indicative of DNA methylation. (B) Patterns of DNA methylation mediated by DNMT3B2 (top) or DNMT3B2 in the presence of DNMT3B4 (bottom) were assessed by bisulfite methylation sequencing. Two independent transfections were analyzed and combined. Closed symbols indicate methylation, open symbols indicate no methylation. While an overall reduction of DNA methylation is clearly observed, the patterns do not appear to have shifted. (TIF) [file pone.0069486.s004.tif]

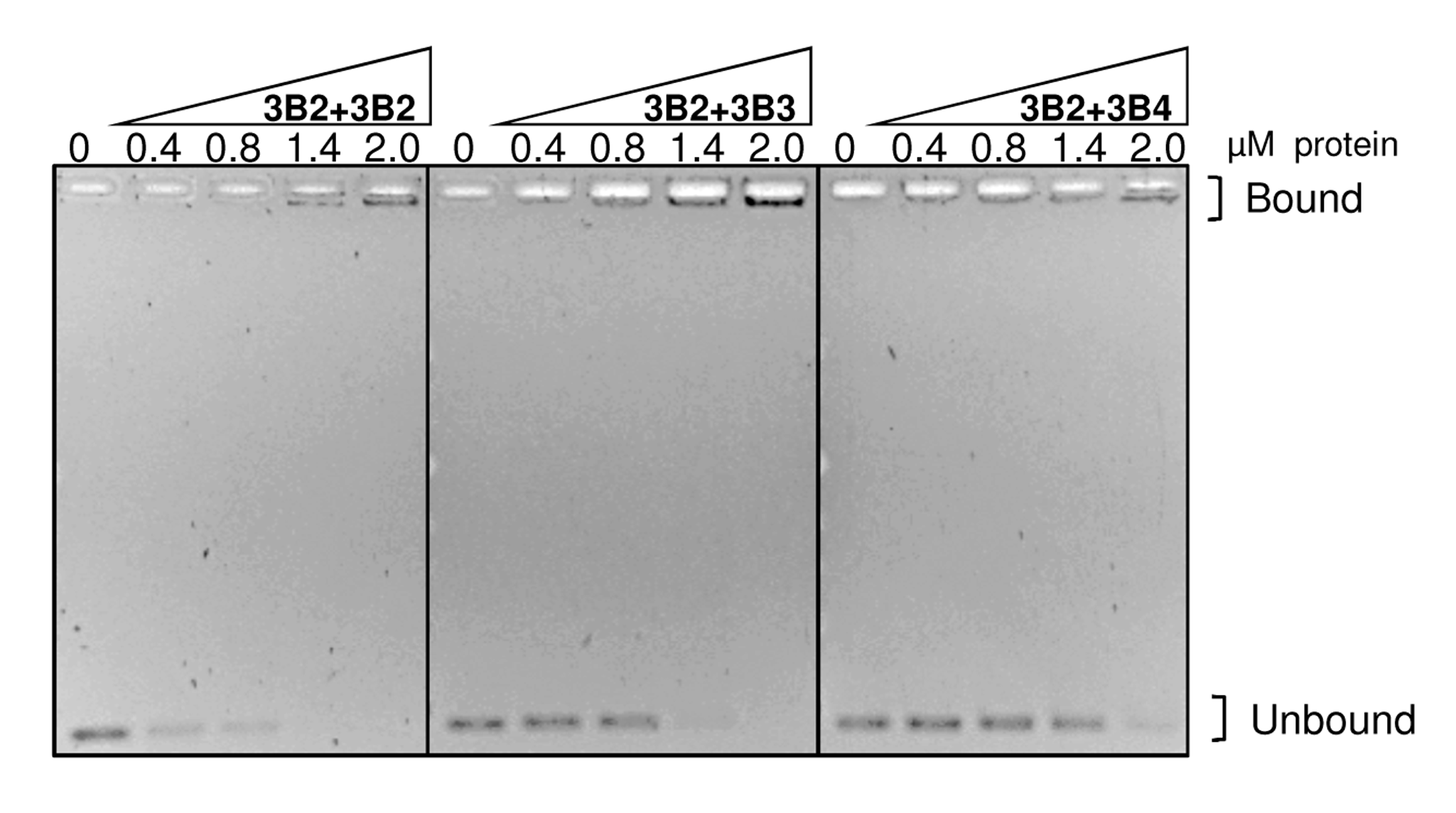

Supplement: Figure S5 — DNMT3B3 and DNMT3B4 hinder DNA binding by DNMT3B2. Representative EMSA gels for full-length DNMT3B2:DNMT3B2, DNMT3B2:DNMT3B3, and DNMT3B2:DNMT3B4 complexes at increasing protein concentrations are shown. A 420 base pair DNA fragment (0.1 µM) was used as a target. (TIF) [file pone.0069486.s005.tif]

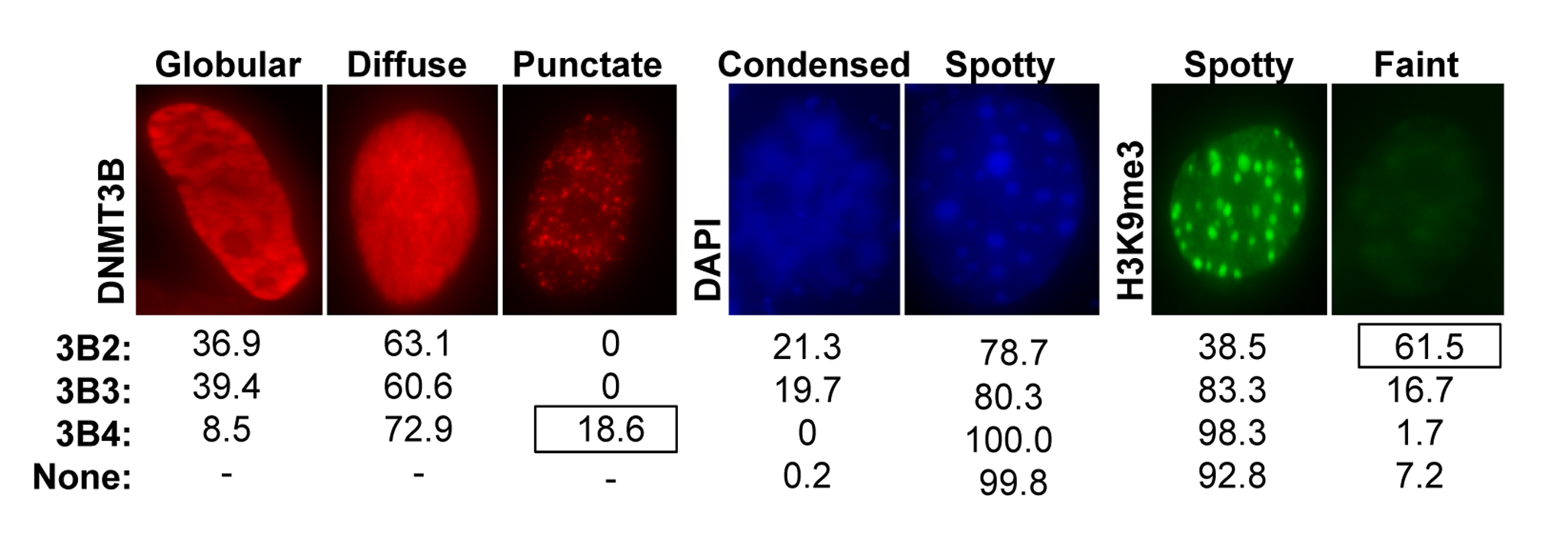

Supplement: Figure S6 — DNMT3B isoforms drive unique and distinct localization, DNA staining, and H3K9me3 patterns in mouse cells. Human FLAG-tagged DNMT3B2, DNMT3B3, or DNMT3B4 were transiently transfected into mouse NIH3T3 cells and immunofluorescence experiments performed with anti-FLAG and anti-H3K9me3 antibodies. See Figure 5 for further details. The total number of independent cells analyzed: DNMT3B2, n = 122; DNMT3B3, n = 66; DNMT3B4, n = 58; none, n = 486. (TIF) [file pone.0069486.s006.tif]

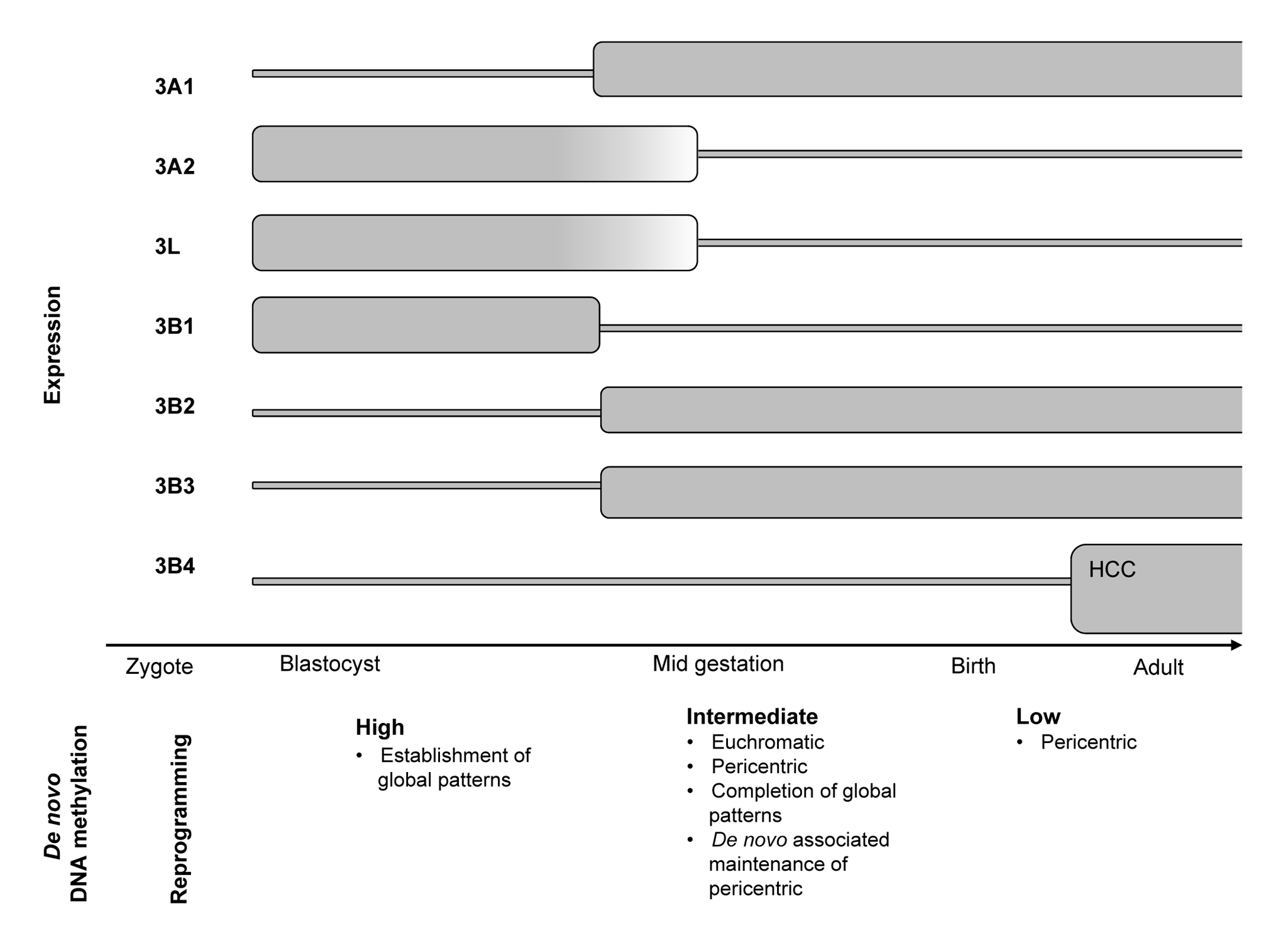

Supplement: Figure S7 — DNMT3 variant expression and de novo DNA methylation during mammalian development. DNMT3A2, DNMT3L, and DNMT3B1 are highly expressed during early development, taking part in establishing global DNA methylation patterns. Upon differentiation, DNMT3B3 becomes highly expressed while DNMT3B1 expression is abruptly shut down. During development, expression of DNMT3A and DNMT3B gradually shifts to DNMT3A1 and DNMT3B2, respectively, while DNMT3L expression is gradually reduced. DNMT3A1, DNMT3B2, and DNMT3B3 take part in completing global DNA methylation patterns. DNMT3B3 is the major splice variant in somatic cells [23] and likely plays a role in directing DNA methylation towards condensed H3K9me3-rich pericentric repeats. Adult tissues generally express low levels of DNMT3 variants and display tissue specific expression of DNMT3B4. In Hepatocellular Carcinomas (HCC) DNMT3B4 becomes highly expressed, and such overexpression is associated with global loss of DNA methylation at pericentric repeats [21]. (TIF) [file pone.0069486.s007.tif]
